# Impact of air humidity on the tenacity of different agents in bioaerosols

**DOI:** 10.1371/journal.pone.0297193

**Published:** 2024-01-26

**Authors:** Paul Siller, Britta Skopeck, Kerstin Rosen, Alexander Bartel, Anika Friese, Uwe Rösler

**Affiliations:** 1 Institute of Animal Hygiene and Environmental Health, Veterinary Centre for Resistance Research–TZR, School of Veterinary Medicine, Freie Universität Berlin, Berlin, Germany; 2 Institute of Veterinary Epidemiology and Biostatistics, School of Veterinary Medicine, Freie Universität Berlin, Berlin, Germany; VIT University, INDIA

## Abstract

Despite the variety of pathogens that are transmitted via the airborne route, few data are available on factors that influence the tenacity of airborne pathogens. In order to better understand and thus control airborne infections, knowledge of these factors is important. In this study, three agents, *S*. *aureus*, *G*. *stearothermophilus* spores and the MS2 bacteriophage, were aerosolized at relative humidities (RH) varying between 30% and 70%. Air samples were then analyzed to determine the concentration of the agents. *S*. *aureus* was found to have significantly lower survival rate in the aerosol at RH above 60%. It showed the lowest recovery rates of the three agents, ranging from 0.13% at approximately 70% RH to 4.39% at 30% RH. *G*. *stearothermophilus* spores showed the highest tenacity with recovery rates ranging from 41.85% to 61.73% with little effect of RH. For the MS2 bacteriophage, a significantly lower tenacity in the aerosol was observed with a recovery rate of 4.24% for intermediate RH of approximately 50%. The results of this study confirm the significant influence of the RH on the tenacity of airborne microorganisms depending on the specific agent. These data show that the behavior of microorganism in bioaerosols is varies under different environmental conditions.

## Introduction

It has long been known that a wide variety of infective agents are transmitted via the airborne route [1]. Aerosols as a significant transmitting medium for pathogens became a focus of attention with the Covid-19 pandemic worldwide. There, airborne transmission of the virus plays a key role in spreading the disease [2]. Infectious severe acute respiratory syndrome coronavirus 2 (SARS-CoV-2) has been detected in the air samples [3] as well as viral RNA [4]. However, few data are available on the factors affecting the tenacity of airborne pathogens [5]. Sedimentation of large particles, temperature, humidity and the UV-C component of solar radiation probably influence the infectivity of airborne microbes in the environment [6–9]. Indoors, relative humidity in particular is subject to large variations with rather constant other environmental conditions such as temperature or UV radiation [10]. The object of this study was therefore to investigate the influence of relative humidity for a representative of bacteria, spores and viruses in comparison under standardized experimental conditions.

The aerosolization process and the detection methods within this study are well standardized and so all data are highly comparable.

We used non- or low-pathogenic agents as surrogate microorganisms because this greatly increased experimental safety and facilitated the generation of large data sets [11], especially when working with aerosols. *Staphylococcus aureus (S*. *aureus)* was chosen as a surrogate primarily for MRSA, but the results may also be extrapolated to other related gram-positive airborne pathogens, e.g. *Streptococcus pneumoniae*. For MRSA, airborne transmission in hospital settings has been presumed for a long time [12] and MRSA has been detected in air samples taken in hospitals [13,14], veterinary clinics [15] and livestock farms [16]. Bacterial spores are very resistant and can thus persist for a very long time under unfavorable environmental conditions and can also be found in the air [17,18]. *G*. *stearothermophilus* was used in this study as a surrogate for spores from gram-positive, aerobic or facultatively anaerobic, pathogens, like *Bacillus anthracis*. The MS2 bacteriophage served as a surrogate for non-enveloped pathogenic viruses because of its characteristics, including a size of 230–300 nm, single-stranded RNA and the absence of a complex tail structure [19]. It is used as a surrogate for viruses causing enteric and respiratory diseases [20]. Some non-enveloped viruses, where airborne spread is an important transmission path, are norovirus, adenovirus or rotavirus in human medicine [21,22] and the foot-and-mouth disease virus in veterinary medicine [23].

This is the first study that allows a direct comparison of the airborne tenacity of three surrogates with entirely different properties at different RH since they were aerosolized using the same methodology.

## Materials and methods

### Experimental design

We used *Staphylococcus aureus* DSM-799 purchased from the German Collection of Microorganisms and Cell Cultures GmbH, *G*. *stearothermophilus* DSM-22 and an MS2 bacteriophage DSM-13767 provided by the Robert-Koch Institute for this experimental series.

### Characteristics of the aerosol chamber

**Fig 1:**
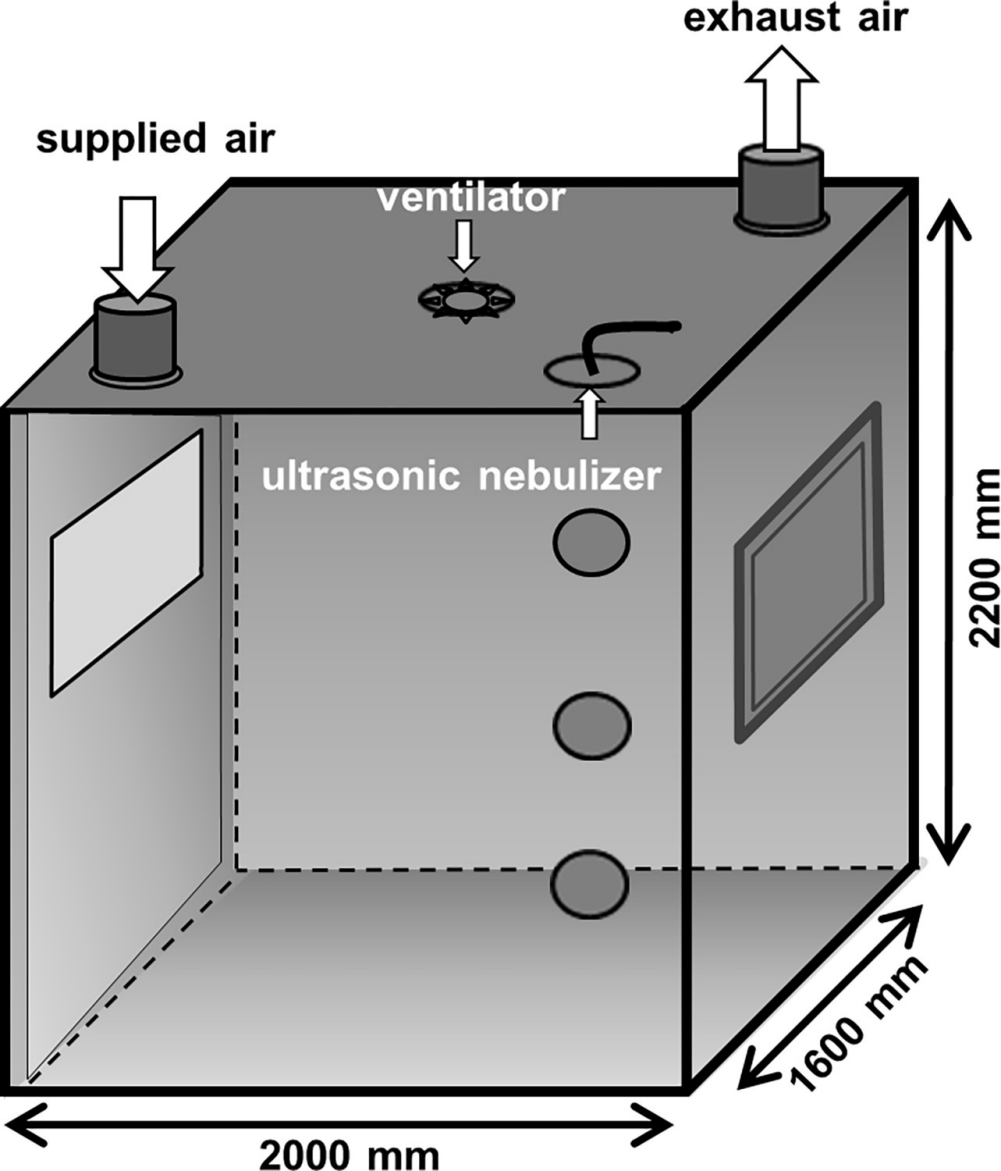
Schematic drawing of the aerosol chamber adapted from Rosen et al. (2018) [24].

The aerosol chamber made of stainless steel has a volume of approximately 7 m^3^ and is used to generate defined bioaerosols (particle number and particle size) under defined adjustable climatic conditions (airflow, RH, temperature). Fig 1 shows a schematic drawing of the chamber, adapted from Rosen et al. (2018). Technical details were also previously published by Rosen et al. (2018) [24]. For all experiments, the airflow was set to 100 m^3^/h and the temperature was set at 24°C. The specific climatic conditions were set in the chamber supply air. All parameters were measured in the exhaust air and automatically documented every minute. Deviations of +/- 5% of temperature and airflow were accepted.

A perfusion pump transported the bacterial, spore and viral suspensions at a rate of 36 ml/h to an ultrasonic nebulizer (SONO-TEK Corporation, Milton, USA) in which droplet aerosols were generated. We used an ultrasonic nozzle with a conical nose for a divergent spray pattern operating at a frequency of 120 kHz, where the average diameter of the droplets generated from the device is initially 18 μm. Thereafter, the droplet size in the aerosol changes very rapidly and shrinks [25]. The collection of air samples began only after the agent had been aerosolized in the chamber for 8 min, allowing for the formation of a stable bioaerosol within the whole chamber volume.

A fan in the ceiling of the chamber dispersed the aerosol. The aerosol chamber is located in a part of a building for experimental studies, where the ambient air is standardized before entering. There, ambient air was filtered using an E 11 filter according DIN EN 1822 and activated charcoal filter before entering the air supply duct in the ceiling of the aerosol chamber. The filter E 11 filters out at least 95% of airborne particles with a size of 0.3 μm and larger. This was installed because there is a cattle and pig barn in the immediate vicinity of the experimental building with the aerosol chamber used within this study and dust-intensive work can therefore occur at certain points with a corresponding increase in particles in the ambient air. The supply air was therefore standardized. The activated charcoal filter was installed to absorb any odors such as ammonia that may occur from the surrounding cattle and pig stables. The airborne particle size distribution and the particle number were monitored at one-min intervals during the 30-min experimental period using an aerosol spectrometer (Grimm, model 1.109, GRIMM Aerosol Technik Ainring GmbH & Co., KG, Germany), which also calculated the aerodynamic diameter. The spectrometer probe was positioned at a point at a height of 1.30 m approximately between the fan and the outlet for the exhaust air.

### Air sampling

Air sampling was performed with three AGI-30 impingers connected in parallel (AGI-30; Neubert Glas GbR, Geschwenda, Germany, VDI Norm 4252–3) at different heights (0.3 m, 0.8 m, 1.3 m) for each experimental replicate. For the air samples from *S*.*aureus* aerosols, the impingers were filled with 30 ml of sterile phosphate-buffered saline (PBS; Oxoid, Wesel, Germany). For the trials with *G*. *stearothermophilus* spores and the MS2 bacteriophage, 30 ml of sterile deionized water was used. The sampling time for each experiment was 30 min. Impingers were connected to vacuum pumps (Leybold S4B; Leybold, Cologne, Germany and Edwards RV3; Edwards, Feldkirchen, Germany). The airflow was approximately 12.5 l/min and was monitored with a rotameter (Analyt, Müllheim, Germany).

### Preparation of the bacterial and viral suspensions

*S*. *aureus* suspensions were prepared individually for each experimental replicate. *S*. *aureus* DSM-799 was incubated overnight in Mueller Hinton broth (Oxoid, Wesel, Germany) with added 6.5% NaCl (MHB+) in a shaking incubator at 37°C and 200 rpm (Multitron, Infors HT, Germany). The following day, 5 ml of this suspension was added to 100 ml MHB+ and incubated for 8h in the shaking incubator. Of this culture, 100 μl were plated on one plate of blood base agar (Blood Agar Base No. 2, Oxoid, Wesel, Germany) for each trial and aerobically incubated for 8 h at 37°C to achieve the exponential growth phase. All colonies on one plate were removed with a plate spreader by adding 3 ml of PBS and homogenized on a vortex mixer for 3 min with glass beads. The suspension was adjusted to the target concentration of 10^9^ colony forming units (cfu)/ml using Mc Farland standard measurement as well as measuring the optical density. Concentrations ranging from 5x10^8^ to 5x10^9^ cfu/ml were accepted for the validity of the experiment.

Cryopreserved vegetative *G*. *stearothermophilus* DSM-22 bacteria were thawed, and streaked to four previously prepared spore agar plates. Ingredients of 1 l spore agar are: 2 g meat extract, 3 g peptone, 0.5 g glucose, 0.36 g CaCl_2_, 0.15 g MgSO_4_x2H_2_O, 0.03 g MgSO_4_xH_2_O and 15 g agar adjusted to a pH-value of 7.0 and solved at 100°C. After streaking the samples on the plates they were incubated for 11 d at 60°C. On days 4 and 7, the bacteria were removed from the agar plates using an inoculation loop and bacteria from each plate were split to two fresh spore agar plates. From day 11 to 15, the agar plates were stored at room temperature, resulting in the sporulation of the bacteria. The spores from the agar plates were suspended in 5 ml of sterile deionized water using a plate spreader. Three washing steps ensued, using centrifugation (2700 g for 15 min) and resuspension. Finally, spores were suspended in 70% ethanol and stored at 4°C. The concentration of the hence achieved stock *G*. *stearothermophilus* spore suspensions was determined by plating serial dilutions on tryptone soy agar (TSA; Oxoid, Wesel, Germany) and incubated at 60°C for 24 h. Spore solutions for aerosolization were diluted in sterile deionized water from the stock solution to the target concentration of 10^7^ cfu/ml for each experimental replicate. Concentrations ranging from 5x10^6^ to 5x10^7^ cfu/ml were accepted for the validity of the experiment.

For the stock solution of MS2 bacteriophage, three colonies of the susceptible host-strain *E*. *coli* DSM-5695 were incubated overnight in 10 ml of Luria/Miller-broth (LB; Roth, Karlsruhe, Germany) at 37°C. The following day, 200 μl of the overnight culture and 100 μl of MS2 bacteriophage suspension were mixed and incubated for 10 min at room temperature. Then 5.5 ml of soft agar was added. Soft agar was prepared by adding bacteriological Agar (Agar No.1; Oxoid, Wesel, Germany) to LB Medium in a ratio of 1:200 and boiling the solution three times. After that, the suspension was transferred to an LB agar plate (Roth, Karlsruhe, Germany) and incubated at 37°C overnight. The following day, the soft agar was removed using a plate spreader and covered with SM buffer (5.8 g NaCl, 2.0 g MgSO_4_x7H_2_O, 50 ml 1 M Tris-HCl in 1 liter H_2_O to pH 7.4) in an Erlenmeyer flask. The mixture was homogenized on a magnetic stirrer (Phoenix instruments, Garbsen, Germany) for 4 h at room temperature, then transferred to a tube and centrifuged (16.000 g, 30 min). Finally, the supernatant was filtered using 22 μm sterile filters (Merck, Darmstadt, Germany) and stored at 4°C until use. Phage solutions for aerosolization were diluted in sterile deionized water from the stock solution to the target concentration of 10^8^ plaque forming units (pfu)/ml for each experimental replicate. Concentrations ranging from 5x10^7^ to 5x10^8^ pfu/ml were accepted for the validity of the experiment.

### Quantification of the suspensions and air samples

Bacterial and spore suspensions used for aerosolization and air samples from the bacteria and spores experiments were quantified by streaking out triplicates of 100 μl of the impinger collection fluid on blood base agar and TSA for *S*. *aureus* and *G*. *stearothermophilus* spores, respectively. The agar plates were incubated for 24 h at 37°C for *S*. *aureus* and 60°C for *G*. *stearothermophilus* spores. Then, cfu were counted and calculated as cfu per ml of impinger fluid. Then it was extrapolated to the final volume of collected liquid in the impinger. This volume was determined using serological pipettes when transferred into sterile tubes. It varied from 18 to 28.6 ml of remaining collection fluid in the impinger after the experiments. Then the cfu in the volume of air collected were calculated and finally the cfu per m^3^ air were specified.

For the quantification of the MS2 bacteriophage, a soft-agar overlay technique was used. Serial dilutions of the phage suspensions and air samples were prepared and 100 μl were incubated with 100 μl of an overnight culture of *E*. *coli* DSM-5695 in triplicates for 10 min at room temperature. Afterward, the fluid was mixed with 5.5 ml of soft-agar, transferred to an LB-agar plate and incubated for 24h at 37°C. Plaques were counted the following day.

### Statistical analysis

All statistical analysis was performed using R version 4.02 (R Foundation Vienna). Since bacterial counts were lognormal distributed, we used the geometric mean for averaging. For statistical analysis, we used a mixed count regression. Due to overdispersion, we choose a negative binomial distribution. For the aerosol chamber experiment, a random effect was used for each of the 72 experiments to account for repeated measures because there were 3 AGI-30 impinger measurements per experiment. We checked for interactions using interaction plots and found an interaction between targeted humidity and the surrogate and an interaction between the impinger height and the surrogate. Both were included in the model as fixed effects. Post-hoc comparisons between all surrogates were adjusted for multiple comparisons using the Bonferroni method. The mixed model was fitted using the R package lme4 (version 1.1–23). Estimated marginal means and multiple comparison post-hoc tests were performed using the emmeans R package (version 1.4.6). Results are reported with 95% confidence intervals. A significance threshold of 0.05 was used. For the visualization, we fitted a restricted cubic spline with the actually measured humidity as a continuous variable using the package mgcv (version 1.8–33).

## Results

**Fig 2:**
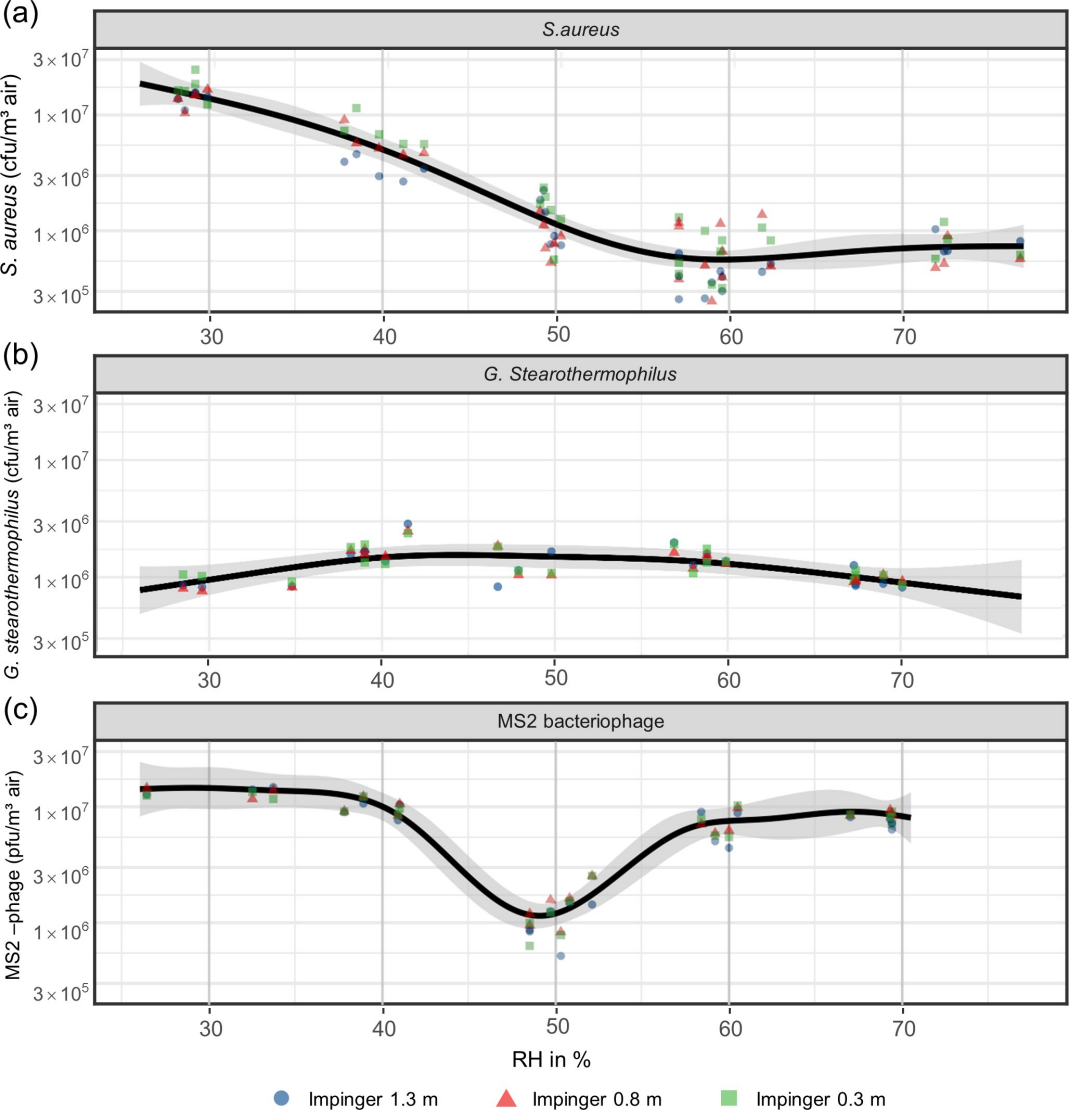
Predicted concentration of *S*. *aureus* (**a**), spores of *G*. *stearothermophilus* (**b**) and MS2 bacteriophage (**c**) per m^3^ at different RH (black line). The symbols represent the raw measurements in cfu or pfu per m^3^ in different impinger heights. The shaded areas indicate the upper and lower 95% confidence interval.

A negative binomial model was used to compare the concentration of surrogates per m^3^ of air for the different targeted RH. All data including raw measurements data points are shown in Fig 2. The exact values of all air samples along with their corresponding humidity can be found in S1 Table.

For *S*. *aureus*, the concentration decreased significantly between each RH level except for 60% and 70% RH where no significant difference was found (p -value = .695). The largest differences were found when comparing the concentrations of approximately 30% RH to 60% and 70% RH, with 25.9-fold (p < .001) and 20.1-fold (p < .001) higher concentration at approximately 30% RH respectively.

The concentration of *G*. *stearothermophilus* spores detected in the air samples was comparatively stable at different RH, with slightly lower concentrations at the lowest (30%) and highest (70%) RH ranges. Significant differences (p < .05) were therefore observed only for the comparisons of 30 to 40%, 30 to 60% and 40 to 70%. Concentrations were 2.0-fold higher at 40% than at 30%, which represented the largest deviation.

For the MS2 bacteriophage, the concentration showed a large drop to ⁓10^6^ CFU per m^3^ air around 50% RH while for all the other RH the concentration was stable at ⁓10^7^ CFU per m^3^ air. Significant differences (p < .001) were detected when comparing the pfu/m^3^ at 50% RH to all other RH ranges. The largest deviation was observed when comparing the concentration per m^3^ at 30% and 40% RH to 50% RH, with an 11.3- fold (p< .001) and 8.3-fold (p < .001) higher concentration, respectively. Another significant but small difference was detected when comparing the pfu/m^3^ at 30% RH and 60% RH range, where a 1.9-fold higher concentration was observed at 30% (p = .003).

Since we also measured the actual humidity in the aerosol chamber for each trial and we expected a continuous effect of the humidity on the agent recovery from the aerosol, a restricted cubic spline was fitted (Fig 2).

When comparing the concentration of surrogates collected at different impinger heights, no significant differences were observed for *G*. *stearothermophilus* spores and the MS2 bacteriophage. However, the concentration of *S*. *aureus* in the lowest impinger (0.3 m) was 1.22 times higher than in the impinger at 0.8 m (p = .003) and 1.44 times higher than in the impinger at 1.3 m (p < .001).

Recovery rates from the aerosol were calculated by dividing the concentration of surrogates detected in the aerosol by the theoretical concentration in the aerosol chamber, which was calculated considering the concentration of the suspensions, the forward speed of the perfusion pump and the airflow measured in the aerosol chamber. The recovery rate of *S*. *aureus* decreased steadily with increasing RH. The recovery rate was highest around 30% RH (4.39%) and lowest at 70% RH (0.13%). For *G*. *stearothermophilus* spores, recovery rates were higher than for *S*. *aureus* at every RH level, ranging from 41.85% at 30% RH to 61.73% at 50% RH. The MS2 bacteriophage showed intermediate recovery rates compared to the bacterial surrogates, ranging from 4.24% at 50% RH to a maximum of 41.57% at 30% RH (Table 1).

**Table 1:** Mean recovery rates in % of the aerosolized surrogates at different RH. N represents the number of measurements per humidity range. Three air samples were simultaneously analyzed per measurement.

	relative humidity				
Aerosolized surrogate	30%	40%	50%	60%	70%
*S*. *aureus*	4.39 n = 5	1.49 n = 5	0.18 n = 6	0.14 n = 10	0.13 n = 4
*G*. *stearothermophilus* spores	41.85 n = 3	43.9 n = 5	61.73 n = 3	48.06 n = 5	50.59 n = 5
MS2 bacteriophage	41.57 n = 3	28.33 n = 4	4.24 n = 6	23.58 n = 4	34.25 n = 4

The mean aerodynamic diameter of aerosolized particles was 3.4 μm for the experiments with *S*. *aureus*, 1.8 μm for the experiments with *G*. *stearothermophilus* spores and 1.4 μm for the experiments with the MS2 bacteriophage.

## Discussion

Knowledge of the tenacity of aerosolized microorganisms under different environmental conditions is useful for assessing the risk of airborne pathogen transmission. However, Tang (2009) pointed out that studies on the tenacity of airborne organisms vary widely in their methodologies, making it difficult to compare results [6].

In the aerosol chamber used for our experiments, microorganism suspensions of defined concentrations are continuously aerosolized into a large volume of air under stable and defined environmental conditions. This dynamic aerosol appears to be advantageous over the use of rotating drums or chambers as described by Goldberg et al. (1958) and recently applied by van Doremalen (2020) [26,27]. The system here appears to be closer to reality when thinking of a person or animal in a room or barn continuously shedding the pathogen via the airborne route.

To the best of our knowledge, this is the first laboratory study to investigate the influence of RH on the tenacity of experimentally aerosolized *S*. *aureus*. We observed the highest recovery rates below a RH of 50%. Madsen et al. (2018) did not find a significant effect of RH on the concentration of airborne *S*. *aureus* in indoor air [28]. However, several studies also indicate a lower tenacity of *S*. *aureus* at high RH. Wilkoff et al. (1969) exposed textiles to airborne *S*. *aureus* at 35% and 78% RH and reported a substantially shorter persistence at 78% RH [29]. Zukeran et al. (2017) investigated the influence of the RH on the inactivation of *S*. *aureus* in an electrostatic precipitator and reported a significantly lower survival rate at RH above 60% [30]. In contrast, bacteria other than *S*. *aureus*, such as gram-negative microorganisms like *Pseudomonas* or *E*. *coli*, showed higher survival rates in air at high humidities in previous studies [31,32]. We think the variation in cell walls between gram-positive and gram-negative bacteria may account for this phenomenon. Gram-positive bacteria possess cell walls with a thick peptidoglycan layer, rendering them less adaptable to fluctuations in water availability. In high-humidity environments, water infiltrates their cells, and they may encounter difficulty expelling the excess water. Consequently, certain bacteria may experience swelling and eventually burst due to these conditions. This highlights the potential of highly varying and individual behavior of different airborne bacteria in the environment. The phenomenon of differential detection of pathogens in bioaerosols under varying environmental conditions was also recently demonstrated in a field study [33]. This underlines the importance of documenting climatic conditions during every air sampling for the prober interpretation of results, both in the experimental setting and during field investigations.

In our study, which is the first to systematically analyze the tenacity of aerosolized *G*. *stearothermophilus* spores at different RH, high recovery rates were measured over a wide range of RH (from 41.85% to 61.73%). For values outside this range, only slightly lower detection rates of the agent in the air were observed. The influence of RH on tenacity in air was lower compared to the other surrogates. This confirms a high tenacity of spores in the aerosolized state. The cell wall of bacterial spores is complex and multilayered, and the water activity has no relevant impact on their ability to survive. This can be an advantage for aerosol studies, where methods, devices, or other factors of air microbial sampling are to be tested, as such a significant influencing factor can be reduced when using spores. Spores of *Bacillus subtilis* subsp. *niger* have been widely used as an indicator in airborne survival studies with bacteria [34,35] and viruses [36] and to validate methods [37,38] due to their extremely high stability in aerosols in several studies.

Concerning the MS2 bacteriophage, the significantly lower recovery rates at an intermediate RH of approximately 50% appear unexpected. However, Verreault et al. (2008) pointed out that the impact of RH on viral infectivity should be determined for each virus individually, as viruses have maximum tenacity at either low, intermediate, or high RH [39]. For the MS2 bacteriophage, the airborne tenacity at different RH has been investigated previously. Dubovi and Akers (1979) aerosolized MS2 bacteriophage from a buffered salt solution at 20%, 50% and 80% RH and showed that recovery was lowest at intermediate (50%) RH and highest at low RH (20%) [19]. This is in line with the results of our study. Trouwborst and De Jong (1973) described the lowest recovery rates of aerosolized MS2 bacteriophage at a RH of 75% [40]. In their study, recovery rates were higher at RHs above and below 75%, which means that they accordingly did not detect a linear correlation between the recovery rate of MS2 bacteriophage and the RH, but instead found the lowest recovery rates at an intermediate RH. Accordingly, for the mycobacteriophage D29, Liu et al. (2012) found the fastest inactivation at intermediate RH (55% ± 5%), followed by high (85% ± 5%) and low (25% ± 5%) RH [41]. The lowest survival rates at intermediate RH have been described for decades for enveloped and non-enveloped viruses. Songer (1967) aerosolized Newcastle disease virus, infectious bovine rhinotracheitis virus, vesicular stomatitis virus, and *Escherichia coli* B T3 bacteriophage at a RH of 10%, 35% and 90% and described the lowest survival rates at 35% RH for all four viruses. It was assumed that initial losses upon aerosolization are highest at low RH, while inactivation over time in the aerosol is highest at high RH [42]. This once again shows that precise and stable adjustment and documentation of relative humidity during experiments with virus aerosols are absolutely necessary and must be taken into account in result interpretation.

We must mention that in addition to the precise recording of microorganisms and environmental conditions, the exact number and distribution of particles in the whole room also plays a major role in the dynamics of bioaerosols. This is a limitation of this study, as the exact distribution of the particles in the different areas of the aerosol chamber was not recorded, but only at one point. In addition, we did not document the particles before aerosolization of the microorganisms in order to assess background levels. However, by pretreating the supply air with an E 11 filter, we assume a certain degree of standardization. Nevertheless, particles in aerosols can behave very differently in distribution depending on their size and the room structure [43] and thus also the microorganisms bound to them. This is particularly important in dusty environments, as is often the case in animal stables. The particle sizes there can vary greatly [44]. For future studies in the aerosol chamber, it is therefore advisable to record the particles before starting aerosolization and, in addition, at different heights and lengths of the chamber in order to be able to evaluate the overall situation with regard to the particles more comprehensively.

In our study, the suspension medium of the investigated agents was different which may also affect the tenacity of the aerosolized microbes [45]. However, we decided to choose the most suitable medium for the specific agent to evaluate the influence of different humidity. *S*. *aureus* was aerosolized from PBS to avoid osmotic shock and associated viability loss [46]. The MS2 bacteriophage was suspended in sterile deionized water because in a preliminary study we observed greatly reduced recovery rates when suspending in PBS. This is consistent with the results in another study in which approximately 30-fold higher survival rates were observed when aerosolizing D29 phage from sterile deionized water instead of PBS [41]. In addition, studies with *E*. *coli* using the same experimental design, also using PBS as the suspension medium, yielded exactly opposite results regarding the influence of air humidity on the infectivity of the bacterial pathogen in the air compared to *S*. *aurues* [32]. Thus, we conclude that the survival rates are attributed more to the pathogen itself rather than the carrier medium.

The discussion of medium used is also interesting in terms of its relevance to a real clinical setting. In such a setting, the pathogen is present in a protein- and cell-rich medium as it is present in various types of saliva or secretions, and subsequently excreted during different activities such as speaking, coughing, or sneezing. Artificially produced saliva substitutes have been used in recent studies to investigate this point further [47]. More complex media in combination with different particle sizes generated first would definitely be interesting starting points for further studies.

## Conclusions

This study confirms that the airborne survival of microorganisms varies greatly depending on the characteristics of the microorganism and environmental factors, in this case RH. This finding should be considered when taking air samples in the field, as the detection limit in air samples can vary depending on the RH. This is also of great importance in experimental studies, as an accurate and stable relative humidity setting must be ensured, especially for bacterial and viral pathogens, in order to obtain comparable study results. In this study, a significantly reduced tenacity of *S*. *aureus* at high RH was described, and *S*. *aureus* showed the lowest recovery rates of the three surrogates. We confirmed high tenacity of *Geobacillus* spores in the aerosol, which was also least influenced by the RH. For the MS2 bacteriophage, a significantly lower tenacity in the aerosol was confirmed for intermediate RH around 50%. Our study highlights that for airborne tenacity studies, appropriate methods, which should be standardized as much as possible, are crucial for comparing the tenacity of different microorganisms.

## Supporting information

S1 Table Concentration of *S*. *aureus*, spores of *G*. *stearothermophilus* and MS2 bacteriophage in cfu or pfu per m^3^ at the specific relative humidity (RH) in the bioaerosol. *pfu/m^3^.
(DOCX)
