# Supplementary material for: Impact of air humidity on the tenacity of different agents in bioaerosols
Source: PLoS One. 2024 Jan 26;19(1):e0297193. doi: 10.1371/journal.pone.0297193 (PMC10817179; doi:10.1371/journal.pone.0297193)
Supplement: S1 Table — *pfu/m3. (DOCX) [file pone.0297193.s001.docx]

| Agent | RH | Impinger 1.3 m | Impinger 0.8 m | Impinger 0.3 m |
| --- | --- | --- | --- | --- |
|  |  | in cfu or pfu/m^3^ | | |
| ***S. aureus*** | 29.2 | 1.57E+07 | 1.52E+07 | 2.52E+07 |
|  | 28.2 | 1.41E+07 | 1.41E+07 | 1.67E+07 |
|  | 28.6 | 1.12E+07 | 1.06E+07 | 1.64E+07 |
|  | 29.9 | 1.47E+07 | 1.69E+07 | 1.26E+07 |
|  | 29.2 | 1.60E+07 | 1.52E+07 | 1.89E+07 |
|  | 42.4 | 3.52E+06 | 4.83E+06 | 5.73E+06 |
|  | 37.8 | 4.04E+06 | 9.26E+06 | 7.46E+06 |
|  | 38.5 | 4.69E+06 | 5.85E+06 | 1.17E+07 |
|  | 39.8 | 3.03E+06 | 5.28E+06 | 6.96E+06 |
|  | 41.2 | 2.73E+06 | 4.62E+06 | 5.77E+06 |
|  | 49.1 | 1.89E+06 | 1.51E+06 | 1.75E+06 |
|  | 49.3 | 2.30E+06 | 1.14E+06 | 2.42E+06 |
|  | 49.4 | 1.49E+06 | 7.25E+05 | 2.01E+06 |
|  | 50.3 | 7.69E+05 | 9.17E+05 | 1.30E+06 |
|  | 49.7 | 7.84E+05 | 5.46E+05 | 1.55E+06 |
|  | 49.9 | 9.25E+05 | 7.96E+05 | 5.79E+05 |
|  | 61.9 | 4.53E+05 | 1.41E+06 | 1.09E+06 |
|  | 58.6 | 2.68E+05 | 5.13E+05 | 1.03E+06 |
|  | 59.6 | 3.09E+05 | 6.83E+05 | 8.48E+05 |
|  | 59.6 | 4.11E+05 | 4.08E+05 | 3.28E+05 |
|  | 62.4 | 5.32E+05 | 5.06E+05 | 8.47E+05 |
|  | 59.5 | 4.59E+05 | 1.18E+06 | 6.79E+05 |
|  | 57.1 | 6.55E+05 | 1.12E+06 | 1.34E+06 |
|  | 57.1 | 2.63E+05 | 3.93E+05 | 5.43E+05 |
|  | 59 | 3.68E+05 | 2.53E+05 | 3.53E+05 |
|  | 57.1 | 4.18E+05 | 1.20E+06 | 4.37E+05 |
|  | 76.8 | 8.35E+05 | 5.88E+05 | 6.36E+05 |
|  | 72.6 | 6.89E+05 | 9.25E+05 | 8.68E+05 |
|  | 72.4 | 6.82E+05 | 5.32E+05 | 1.22E+06 |
|  | 71.9 | 1.06E+06 | 4.92E+05 | 5.87E+05 |
| ***G. stearothermophilus* spores** | 34.8 | 8.37E+05 | 8.23E+05 | 9.16E+05 |
|  | 28.5 | 8.56E+05 | 8.03E+05 | 1.06E+06 |
|  | 29.6 | 8.23E+05 | 7.65E+05 | 1.02E+06 |
|  | 38.3 | 1.58E+06 | 1.69E+06 | 1.81E+06 |
|  | 39 | 1.65E+06 | 1.73E+06 | 1.91E+06 |
|  | 41.5 | 2.85E+06 | 2.47E+06 | 2.39E+06 |
|  | 39 | 1.66E+06 | 1.58E+06 | 1.33E+06 |
|  | 40.2 | 1.37E+06 | 1.50E+06 | 1.30E+06 |
|  | 46.7 | 8.30E+05 | 1.84E+06 | 1.83E+06 |
|  | 47.9 | 1.14E+06 | 1.05E+06 | 1.13E+06 |
|  | 49.8 | 1.66E+06 | 1.04E+06 | 1.09E+06 |
|  | 59.9 | 1.37E+06 | 1.31E+06 | 1.36E+06 |
|  | 58 | 1.27E+06 | 1.19E+06 | 1.09E+06 |
|  | 56.9 | 1.98E+06 | 1.63E+06 | 1.92E+06 |
|  | 58.8 | 1.41E+06 | 1.47E+06 | 1.34E+06 |
|  | 58.8 | 1.59E+06 | 1.55E+06 | 1.74E+06 |
|  | 67.4 | 8.48E+05 | 9.57E+05 | 1.04E+06 |
|  | 67.4 | 1.26E+06 | 9.15E+05 | 9.39E+05 |
|  | 67.4 | 8.75E+05 | 9.28E+05 | 1.14E+06 |
|  | 69 | 8.83E+05 | 1.04E+06 | 1.05E+06 |
|  | 70.1 | 8.22E+05 | 9.23E+05 | 8.70E+05 |
| **MS2 bacteriophage*** | 26.4 | 1.31E+07 | 1.49E+07 | 1.29E+07 |
|  | 32.5 | 1.44E+07 | 1.20E+07 | 1.38E+07 |
|  | 33.7 | 1.51E+07 | 1.42E+07 | 1.20E+07 |
|  | 37.8 | 9.19E+06 | 9.36E+06 | 9.33E+06 |
|  | 40.9 | 7.85E+06 | 8.61E+06 | 8.74E+06 |
|  | 41 | 1.07E+07 | 1.09E+07 | 9.94E+06 |
|  | 38.9 | 1.09E+07 | 1.25E+07 | 1.26E+07 |
|  | 49.7 | 1.28E+06 | 1.61E+06 | 1.25E+06 |
|  | 50.8 | 1.52E+06 | 1.66E+06 | 1.57E+06 |
|  | 48.5 | 8.84E+05 | 9.69E+05 | 6.44E+05 |
|  | 52.1 | 1.47E+06 | 2.60E+06 | 2.59E+06 |
|  | 50.3 | 5.28E+05 | 8.48E+05 | 8.01E+05 |
|  | 48.5 | 8.58E+05 | 1.22E+06 | 1.02E+06 |
|  | 60 | 4.53E+06 | 6.39E+06 | 5.63E+06 |
|  | 59.3 | 5.17E+06 | 6.03E+06 | 5.79E+06 |
|  | 58.4 | 9.30E+06 | 7.25E+06 | 8.05E+06 |
|  | 60.5 | 9.07E+06 | 1.01E+07 | 1.06E+07 |
|  | 69.4 | 6.52E+06 | 9.00E+06 | 7.31E+06 |
|  | 69.4 | 7.39E+06 | 8.59E+06 | 9.31E+06 |
|  | 67 | 8.36E+06 | 8.62E+06 | 8.77E+06 |
|  | 69.3 | 8.01E+06 | 9.59E+06 | 8.39E+06 |

S1 Table: Concentration of *S. aureus*, spores of *G. stearothermophilus* and MS2 bacteriophage in cfu or pfu per m³ at the specific relative humidity (RH) in the bioaerosol.

*pfu/m^3^
